# Extra-Ribosomal Roles for Ribosomal Proteins and Their Relevance to Tumour Suppression, Carcinogenesis and Cancer Progression

**DOI:** 10.3390/cancers17172825

**Published:** 2025-08-29

**Authors:** Sreepriya Pk, Joshua Freimanis, Thomas Kovala, Amadeo M. Parissenti

**Affiliations:** 1 Ph.D. Program in Biomolecular Sciences, Laurentian University, Sudbury, ON P3E 2C6, Canada; spk@laurentian.ca (S.P.); tkovala@nosm.ca (T.K.); 2 Biomedical Biology Program, School of Natural Sciences, Laurentian University, Sudbury, ON P3E 2C6, Canada; jfreimanis@laurentian.ca; 3 Division of Medical Sciences, Northern Ontario School of Medicine, Sudbury, ON P3E 2C6, Canada; 4 Health Sciences North Research Institute, Sudbury, ON P3E 2C6, Canada; 5 Rna Diagnositcs, Inc., Toronto, ON M4T 1L9, Canada

**Keywords:** ribosomal proteins, extra-ribosomal roles, tumour suppression, carcinogenesis, tumour progression

## Abstract

Protein translation by ribosomes is one of the most energetically costly cellular processes. Consequently, the number and activity of ribosomes in cells and tissues are precisely tailored to match metabolic demands. While ribosomal proteins (RPs) play essential roles in facilitating and regulating the translation of mRNA transcripts into protein, there is increasing evidence that free RPs not bound to ribosomes can play important roles in cellular regulation. Often, free RPs act as tumour suppressors by multiple mechanisms, for example, by inducing cell cycle arrest through their ability to bind and inhibit MDM2-mediated p53 degradation. Dysregulation of these RPs, however, can result in various diseases like Diamond-Blackfan anemia, ribosomopathies, and other diseases. In cancer, epigenetic modifications, altered transcription, and processing defects in the rRNAs create “onco-ribosomes” that strongly support tumour cell replication, invasion and metastasis. In this context, free RPs in tumour cells (often mutated or post-translationally modified) further promote tumour cell proliferation, invasion, and metastasis. This review focuses specifically on extra-ribosomal roles for RPs, where depending upon cellular context, they act outside of the ribosome to either suppress tumorigenesis in normal tissues or promote tumour proliferation and progression. This new understanding of the interplay between RPs and pathways suppressing or promoting tumorigenesis further emphasizes why the ribosome is increasingly being seen as an important therapeutic target in human cancers.

## Simple Summary

Ribosomes are large macromolecular organelles composed of ribosomal proteins and nucleic acids that facilitate the translation of mRNA transcripts into proteins within mammalian cells. It was once believed that ribosomal proteins acted solely within ribosomes to help stabilize the organelle’s structure and facilitate protein synthesis. However, since 2004, it has become increasingly clear that during stress ribosomal proteins can act outside of the ribosome as tumour suppressors through effects on p53 and other proteins. In contrast, ribosomal proteins unbound to ribosomes can strongly promote tumour cell proliferation and disease progression in cancer patients. This review highlights recent advances in our understanding of the role of many ribosomal proteins in both tumour suppression and tumour promotion, largely contingent on cellular context.

## 1. Introduction

### 1.1. Carcinogenesis and Treatment of Human Cancers

Cancer remains a significant global health concern and one of the leading causes of death according to the World Health Organization (WHO). However, due to improvements in disease diagnosis, early detection, prevention strategies, and treatment options in both the adjuvant and neoadjuvant settings, there has been a notable decline in overall cancer mortality. Nevertheless, the International Agency for Research on Cancer predicts that 35 million new cancer cases will be diagnosed by 2050. Thus, there remains a rapidly growing cancer burden on society, due to an aging population and a combination of both genetic and environmental factors that permit cells to escape mechanisms that normally control cell proliferation and migration [1,2,3]. A series of complex genetic and molecular changes occurs during tumorigenesis, typically involving disruptions in cellular pathways involved in cell replication, cell invasion, and programmed cell death. Several factors like tobacco smoking, exposure to radiation and carcinogens, diet, family history, and exposure to viruses can all impact on cancer incidence [4,5,6,7,8]. Particular emphasis has been placed on how specific exposures to viruses, radiation, or carcinogens results in the activation of protooncogenes or the inactivation of tumour suppressor genes [9].

### 1.2. Role of Oncogenes and Tumour Suppressor Genes in Tumorigenesis

Increased expression or activity of oncogenes such as *c-MYC* [10], *KRAS* [11], *BRAF* [12], and *HER2* [13] or inactivation of tumour suppressor genes such as *P53* [14], *BRCA1*, and *BRCA2* [15], can lead to uncontrolled cell division and the inhibition of cell death pathways [16,17]. Growth factors and cell signaling proteins such as AKT, NF-kB, and HSP70 can also promote tumorigenesis [18,19,20] by stimulating tumour angiogenesis [21] and activating telomerase [22] and other proteins that promote tumour cell invasion and metastasis [23].

The level and activity of ribosomes in mammalian cells are closely tied to their cellular metabolic requirements and environmental conditions [24]. Thus, ribosomal activity is tightly regulated through complex mechanisms involving signaling pathways like mTOR (mechanistic target of rapamycin) which integrates nutrient availability and metabolic requirements with protein synthesis [25]. Thus, cancer cells have high ribosome numbers and activity to support the level of protein synthesis required for cell proliferation, invasion and survival [26,27,28].

### 1.3. The Critical Role of Protein Synthesis in Cancer Development

The elevated levels of protein synthesis in cancer cells are often due to mutations and epigenetic changes in genes that affect ribosome biogenesis and function. Such gene modifications lead to the formation of ribosomes that are tailored to support continuous cell replication and invasion (the “onco-ribosome”) [29]. Such ribosomes preferentially translate mRNAs encoding oncogenic proteins, such as c-Myc, Bcl-2, survivin, and cell cycle regulators. These mRNAs are mostly translated by ribosomes that contain RACK1 (receptor for activated kinase 1), especially during stress. They have a 5′ untranslated region (5′UTR) that contains an internal ribosome entry site (IRES) element, which enhances the translation of these proteins [30,31]. The role of onco-ribosomes is particularly evident in cancer metastasis, a multistep process driven by proteins such as matrix metalloproteins (MMPs) [32], epithelial–mesenchymal transition (EMT) factors [33,34], and cytoskeletal remodeling proteins [35]. The aberrant higher ribosome activity ensures overproduction of these proteins, enabling cancer cells to degrade the extracellular matrix, invade nearby tissues, and migrate to secondary sites [36]. Moreover, the human ribosomal protein (RP) genes exist as paralogous pairs—genes that have a common ancestral origin through gene duplication but have evolved to provide similar but not identical functions. Ribosomes that incorporate specific paralogs of RPs are functionally distinct, leading to translation of specific subsets of mRNAs. Cancerous cells selectively translate mRNAs that drive tumour cell survival, proliferation and invasion [26,37,38,39,40,41]. Tumour metastasis remains a leading cause of disease recurrence and cancer-related deaths, underscoring the importance of understanding ribosomal dysregulation in cancer biology [42,43].

### 1.4. Ribosome Structure and Function

The ribosome is a macromolecular complex of ribosomal RNAs (rRNAs) and proteins. Ribosome numbers and activity are maintained through a balance of ribosome biogenesis, quality control, recycling, and ribosome degradation processes [44,45,46]. Eukaryotic ribosomes are made up of 2 subunits containing 4 different rRNAs. The ribosomes are transcribed from rRNA genes by RNA polymerases I and III in the nucleolus of cells. The large and small subunits of ribosomes contain approximately 80 different RPs, which are synthesized in the cytoplasm and imported into the nucleus by importins, which recognize nuclear localization signals on RPs [47,48]. The ribosomes are then assembled within the nucleus. The RPs are distributed among the ribosomes’ 60S large subunit (LSU) and 40S small subunit (SSU). The eukaryotic 60S subunit consists of 47 RPs and the 5S, 28S and 5.8S rRNAs, while the 40S subunit consists of the 18S rRNA and 33 RPs [49,50]. The catalytic activity of the ribosome is carried out by the rRNAs, while the RPs on the ribosome surface help stabilize its structure [51]. Ribosomes can exist in cells as free ribosomes within the cytoplasm or complexed to intracellular membranes within the rough endoplasmic reticulum. Several ribosomes move along a single messenger RNA (mRNA) chain at one time as polysomes, each reading a specific sequence and producing a corresponding protein molecule. Ribosomes also serve as docking stations for transfer RNAs (tRNAs), which have anti-codons that recognize codons within mRNAs [52]. Primary transcripts encoding proteins are processed into mRNAs, which then emerge from the nucleus and bind to the smaller subunit of the ribosome via their 5′UTR. Once the SSU identifies the start codon, the binding of the anti-codon for the methionine tRNA then permits the LSU to bind, resulting in the formation of the complete 80S ribosome. In the decoding center of the ribosome, the anti-codons for the appropriate tRNAs then bind to the codons within the mRNA, permitting the peptidyl transferase center to continue to add amino acids to the growing peptide. This continues until the synthesis of the new protein is complete. When the ribosome recognizes a stop codon (UGA/UAA/UAG), both subunits dissociate from each other, and the newly synthesized peptide is released [53].

Protein synthesis is regulated by numerous proteins, including eukaryotic initiation factors (elFs), elongation factors (eEFs), and release factors (eRFs). Several miRNAs and RNA binding proteins are also involved in this translation process [53,54,55]. In addition to the classical mRNA translation pathways that are 5′cap dependent, IRES-dependent mRNA translation can also occur under conditions of environmental stress to permit translation of specific mRNAs that promote cell survival [42,56,57]. Cellular stressors like hypoxia, nutrient deprivation, oxidative stress, or a viral infection can induce IRES-mediated translation in cells. The initiation of IRES-mediated translation is associated with several pathological outcomes. In cancer cells, IRES-mediated translation can be hijacked to ensure the continued synthesis of proteins that promote tumour proliferation, even when global protein synthesis is reduced due to ribosomal or nucleolar stress (cellular stress that disrupts ribosome biogenesis) [42,56].

### 1.5. Translation Quality Control Mechanisms

Despite being a highly regulated and complex process, errors can occur at any step during mRNA translation, resulting in the production of truncated proteins that can be detrimental to cell function. Thus, the surveillance pathway associated with ribosome quality control is critical to ensure the quality and fidelity of protein translation. The ribosome-associated quality control (RQC) mechanism identifies stalled ribosomes during protein synthesis [31,58,59]. The No-Go decay (NGD) pathway specifically identifies stalled ribosomes caused by truncated mRNAs and cleaves and removes the mRNAs from the stalled sites [60]. Non-stop decay (NSD) targets and degrades mRNAs that lack stop codons, leading to the release of the stalled ribosome [61]. The nonsense-mediated decay (NMD) pathway monitors and degrades mRNAs with premature stop codons [62]. RNA surveillance mechanisms involving the exosome pathway degrade defective rRNAs in the nucleus and cytoplasm. For example, the 28S and 18S non-functional rRNA decay (NRD) pathways identify and degrade defective rRNAs that could impair translation fidelity [63].

A cell needs to quickly detect and adapt to cellular stressors (such as nutrient deprivation or genotoxic/proteotoxic stress). Since ribosomes are the site of protein production (the most energetically costly process in cells), the number of ribosomes and protein production is tailored to meet the energy needs of the cell. During ribosomal stress (impaired ribosome production), two main protein quality control pathways are activated to ensure cellular energy balance. The first pathway is the ubiquitin–proteasome system (UPS) [64,65] that identifies misfolded proteins that are tagged with ubiquitin for subsequent degradation by the proteasome. In addition, misfolded proteins on the ribosome during conditions of ribosomal stress are tagged with ubiquitin, leading to the degradation of entire ribosomes through a selective autophagic process called ribophagy [66,67,68].

### 1.6. Modifications of rRNAs and RPs

Post-transcriptional modifications of rRNAs and post-translational modifications of RPs are critical for normal ribosome function. rRNA modifications (such as the 2′-O-methylations present in 95% of rRNAs [69], pseudo uridylations [70], and nucleotide modifications [71]) play important roles along with the RPs in maintaining the structural integrity and functional efficiency of ribosomes. These modifications occur at important sites within the rRNAs that play critical roles in protein translation, including the peptidyl site, the decoding site and the subunit interface of the ribosome [72]. Therefore, any alterations in these modifications could easily alter ribosome function, resulting in reduced or enhanced protein synthesis.

Dysregulation of rRNA modifications is known to contribute to specific disease states, including cancer. For example, hyper- and hypomethylations of specific sites within rRNAs are found in tumour cells [69,72,73]. Furthermore, the loss of a single modification in the 28S rRNA can promote the specific translation of proteins associated with Wnt/β-catenin signaling, a pathway that promotes cell migration and proliferation [74]. Human cancers often exhibit specific changes in the methylation of the 18S and 28S rRNAs, enhancing the translation efficiency of mRNAs that have optimal codons for amino acid transporters [75]. This results in a translational bias that supports increased amino acid metabolism, a hallmark of leukemic stem cells [76]. Such metabolic reprogramming of the ribosome is crucial for the survival and proliferation of tumour cells, facilitating enhanced amino acid transport and utilization. Fibrillarin (FBL) [77], a snoRNP 2′-o-methytransferase that plays a role in rRNA methylation, is highly expressed upon malignant transformation of cells. In normal cells, the expression of FBL is directly regulated by p53, which acts as a repressor of FBL protein expression, thereby preventing abnormal rRNA methylations. Loss of *P53* function leads to increased FBL activity, resulting in rRNA modifications that promote IRES-mediated translation of mRNAs for proteins such as IGF-IR and c-Myc. These proteins are known to be associated with tumour initiation, tumour cell proliferation, colony formation and chemotherapy resistance [78]. In patients with breast cancer, there is significant intra-patient and inter-patient variability in the 2′-O-methylation of rRNAs [75]. This variability is associated with differences in tumour grade and subtype, suggesting a potential role for rRNA 2′-O-methylations in disease heterogeneity. In conclusion, alterations in the sequence or modifications of rRNAs can alter ribosome function and stability, permitting them to function as onco-ribosomes that preferentially translate transcripts that promote the survival and function of tumour cells, including those associated with cell cycle regulation, anti-apoptotic pathways, angiogenesis, metastasis and cell invasion [70,79,80,81,82].

## 2. Alterations in the Expression, Post-Translational Modification, and Function of RPs

### 2.1. Alterations in RP Expression in Cancer and Under Stress

It is becoming increasingly apparent that RPs are not simply structural components of the ribosome, nor are they solely involved in the translation of mRNAs. Rather, many RPs have extra-ribosomal functions that impact on cellular processes directly related to tumorigenesis. The overexpression of RPs can occur in pathologic conditions and elevated expression of RPs is often seen in cancer cells compared to their non-cancerous counterparts [83]. Generally, in healthy unstressed cells, RPs not incorporated into ribosomes are rapidly degraded by proteasomes after ubiquitination by the Tom1 ubiquitin E3 ligase [84]. Similarly, during cellular stress such as nutrient deprivation, some RPs are degraded upon activation of ribophagy. However, other RPs escape degradation and play extra-ribosomal roles during stress, such as exposure to DNA damaging agents or during nutrient limitation. They then impact a variety of cellular processes, including cell survival, cell cycle progression, apoptotic pathways, and cell differentiation [43,85,86,87]. For example, Sirozh et al. demonstrated that nucleolar stress in mice caused an accumulation of free RPs, ultimately contribute to accelerated aging [88]. Additionally, cellular stresses such as nutrient deprivation, oxidative stress, and exposure to DNA damaging agents have the potential to inhibit global protein synthesis and hence affect ribosome assembly and function.

### 2.2. Post-Translational Modifications of RPs During Stress

Depending upon the nature of stress and the specific cellular context, certain post translational modifications occur in RPs. These include RP phosphorylation, carbonylation, glutathionylation, sumoylation, glycation, glycosylation, acetylation, methylation, nitration, neddylation and ubiquitination [87,89,90,91,92,93,94,95]. Among these, the most widely explored post-translational modifications of RPs in cancer are phosphorylation [96,97] and ubiquitination [28,31].

Phosphorylation of RPs can control their activity and influence the rate of mRNA translation. They can also induce RPs to play extra-ribosomal roles, such as the activation of DNA damage response pathways (see sections on RPS3 and RPS6). Protein ubiquitination is one of the most important post-translational modifications of proteins, in which one or more ubiquitin (Ub) molecules are covalently attached to one or more lysine residues within the protein [90,92,98]. Depending upon the lysine residue to which Ub is linked, a protein can have distinct outcomes. The ubiquitination of a protein can mark it for degradation, or it can facilitate or inhibit protein–protein interactions. These can result in a change in protein function or localization—strongly impacting on specific cellular processes. Typically, free RPs are ubiquitinated by a E3-Ub-ligase and are degraded by the proteasome. However, depending on the lysine residues ubiquitinated, the RPs may escape degradation and participate in protein–protein interactions that impact on numerous cellular functions, including DNA repair activity, cell cycle regulation, and apoptosis. In cancerous cells, the ubiquitination of specific RPs can promote tumour invasion, tumour metastasis, and resistance to chemotherapy treatment, thereby contributing to tumour progression (see sections on RPL5, RPL11, RPS3, and RPL23a).

### 2.3. Contribution of Mutations in RPs to Tumorigenesis

Whole-genome sequencing, exosome sequencing, and transcriptomic profiling studies have provided critical insight into the roles that mutations in RPs play in human cancers [99,100,101]. Specifically missense mutations, heterozygous deletions, point mutations, and frame shift mutations in RP genes frequently occur in many cancer types. A large-scale study of various cancers using the cancer genome atlas identified mutations and copy number alterations in RP genes that were consistent across tumour types [43,99]. Mutations in RP genes in cells can result in reduced or elevated levels of specific RPs [102]. These alterations can cause irregular activation of cell cycle check points through the effect of these RPs on the p53 tumour suppressor pathway (see below), ultimately promoting oncogenesis. Genetic mutations in RPs are also associated with ribosomopathies like DBA, Shwachman–Diamond syndrome, and 5q-syndrome. Patients with these defects have a 5-fold higher lifetime risk of developing cancer than the general population [103,104]. Apart from cancer, mutations in RP genes are also associated with neurodegenerative diseases, developmental diseases, autoinflammatory and immune diseases, and cardiomyopathies [43,105].

### 2.4. RPs Regulating the P53-MDM2 Pathway

There is now significant evidence for extra-ribosomal roles for RPs, particularly in the regulation of the P53-mouse double minute 2 (MDM2) pathway under conditions of ribosomal stress [99,106,107,108,109]. The tumour suppressor gene *P53* induces cell cycle arrest at key check points to prevent replication of damaged DNA. The involvement of p53 in the regulation of a whole host of important cellular processes like DNA repair, cell cycle arrest, apoptosis, senescence, and inhibition of angiogenesis underscores its critical role in cellular homeostasis and tumour suppression. Under normal conditions, p53 is maintained at a low level in cells through the activation of MDM2, an E3 ubiquitin ligase that ubiquitinates p53 and targets it for degradation via the 26S proteasome [110,111]. However, under cellular stress, some RPs (for example, RPL37, RPS15, and RPS20) bind to MDM2, blocking its ability to ubiquitinate p53 for subsequent degradation [112]. Similarly, RPS4X binds to and suppresses both MDM2 autoubiquitination and SCF complex-mediated ubiquitination of proteins, including p53 [113]. This permits levels of p53 to accumulate in cells, enabling it to promote cell cycle arrest and DNA repair. Should the stress be prolonged, p53 can then promote cellular senescence or apoptosis. Several other studies support the role of RPs in binding to MDM2 to block p53 ubiquitination and degradation [106,114,115,116]. Thus, it is not surprising that specific mutations in the above RPs block their ability to up-regulate p53 levels and induce p53-mediated tumour suppression. Consistent with this view, missense mutations in RPS20 blocks its ability to bind to MDM2. These RP mutations are linked to DBA, which as stated above, is linked to higher cancer incidence [103,104]. A number of other studies have also shown the ability of RPs to bind MDM2 to prevent p53 degradation, although some RPs can act in a contrary manner [107,108,117].

### 2.5. Role of RPs in Oxidative Stress

Mammalian cells have a variety of mechanisms to protect themselves from oxidative stress, including cellular enzymes that neutralize reactive oxygen species (ROS). These include superoxide dismutase, catalase, and glutathione peroxidase (reviewed in [118]). However, under prolonged stress, these antioxidant enzymes become depleted, resulting in oxidative damage to DNA, RNA, lipids, and proteins [119,120]. Damage to RPs can result in RP misfolding and complete loss of RP function. Specific stressors can promote the involvement of select RPs in extra-ribosomal roles that activate metabolic pathways that support a cell’s ability to cope with stress. These typically are tumour-suppressive roles for RPs. However, depending upon the cellular context, such pathways regulated by RPs may also promote tumour cell proliferation. The following section describes extra-ribosomal roles for RPs during cellular stress, with particular emphasis on cells that have undergone malignant transformation (cancerous cells).

## 3. Extra-Ribosomal Roles for Specific RPs in Normal and Tumour Cells

### 3.1. RPs L5 and L11

The LSU RPs L5 and L11 are critical components of the ribosome, with well-established roles beyond protein translation. Like RPs L37, S15, and S20 (see above), RPs L5 and L11 also play a role in the regulation of MDM2 activity. Studies have shown that RPL5 and RPL11 can bind to the 5S rRNA and form a small ribonucleoprotein (5S RNP) complex [121]. The 5S rRNA is located at the central protuberance of the LSU and RPL11 is found to be in contact with the head domain of the 40S smaller subunit, acting as a bridge between the two subunits. The 5S RNP can then form a complex with MDM2 [109,117,122,123,124]. RPs L11 and L5 are very basic proteins and bind directly to the central acidic domain of MDM2, which is a critical site for the binding of p53 [117]. This binding is abolished when mutations are introduced into the coding region for the central acidic region [106,117]. Furthermore, mutations in RPL11 have been found to hinder its ability to interact with MDM2 [125]. The binding of these RPs to MDM2 causes a change in the structure of MDM2, inhibiting its E3 ligase activity. This prevents the formation of ubiquitinated p53, resulting in less p53 being targeted for proteolytic degradation and consequently increased p53 levels. The higher levels of p53 then permit the protein to induce cell cycle arrest by promoting transcription of the cyclin-dependent kinase (CDK) inhibitor CDKI or by promoting apoptosis (reviewed in [126,127], respectively). Although MDM2 is an E3 ligase, the binding of RPL11 or RPL5 does not lead to their ubiquitination and degradation. In fact, the binding of these RPs to MDM2 induces a conformational change that protects them from degradation. The overexpression of RPs (beyond that required for protein translation) has been shown to inhibit MDM2-mediated p53 degradation. During nuclear stress, RPL11 helps to increase the translation of its own mRNA, thereby enhancing its interaction with MDM2 in order to support elevated p53 levels and activity [109]. In contrast, downregulation of RPL5 and RPL11 results in decreased cellular p53 levels, even under unstressed conditions [121,128]. This helps to illustrate the critical extra-ribosomal roles that these RPs play in promoting p53-mediated tumour suppression in cells during stress.

Apart from regulating cellular p53 levels, both RPs L11 and L5 regulate c-Myc gene expression during ribosomal stress (also known as nucleolar stress) [129,130]. C-Myc is an oncoprotein that plays a crucial role in cell proliferation, growth, differentiation and apoptosis. It can accelerate cell proliferation by promoting the expression of cyclins D1 [131] and E [132] (genes required for the G1-to-S phase transition during cell cycle progression). This permits cells to bypass the G1 check point and enter the S phase of the cell cycle, promoting rapid cell division. However, in normal tissues, c-Myc promotes cell proliferation only when there is a need for tissue repair. During nutrient stress and hypoxia, c-Myc also helps to alter cellular metabolism towards glycolysis, which generates critical cellular metabolites to promote cell replication [133]. The level of c-Myc in cells is strictly kept at a low level in normal tissues, as c-Myc also permits cells to evade apoptosis and continue cell division, even in the presence of DNA damage [134]. Unregulated, this promotes tumorigenesis. Thus, there are mechanisms by which specific RPs suppress c-Myc expression.

Upon ribosomal stress, RPs L11 and L5 (uncomplexed with ribosomes) bind to the 3′UTR of the c-Myc mRNA along with two subunits of the RNA-induced silencing complex (RISC). This permits suppression of c-Myc expression through the RISC-mediated miRNA sequestration mechanism [129]. RPs L11 and L5 can also promote apoptosis in the absence of p53. It has been shown that both these RPs can compete with MDM2 for binding to the N-terminus of TAp73, a p53 homolog that is capable of transactivating critical genes involved in cell cycle arrest and apoptosis, such as Bax, Puma, and P21 [135]. Binding of these RPs to TAp73 inhibits the binding of MDM2 to TAp73, thus protecting it from the proteasomal degradation by MDM2 [135].

RPs L5 and L11 have strong implications for cancer treatment outcome. RPL11 overexpression in gastric cancer patients treated with 5-fluoro uracil (5-FU) is associated with good prognosis [136]. Further studies indicated that 5-FU suppresses tumour progression through activation of the RPL11-p53 pathway [136]. Another role for RPs L5 and L11 in cancer is related to their association with MeCP2 (Methyl-CpG-binding protein), a protein that epigenetically regulates gene expression by binding to methylated CpG sites within genes [137]. MeCP2 expression is significantly higher in breast cancer [137] and is associated with disease progression and poor prognosis in several cancers. Recent studies have shown that MeCP2 suppresses RPL11 and RPL5 transcription, promoting p53 degradation, tumour cell proliferation and inhibition of apoptosis [137]. In contrast, the knockdown of MeCP2 expression restores cellular levels of RPs L11 and L5, thereby inhibiting cell proliferation and migration and inducing cell apoptosis.

Despite the above extra-ribosomal roles for RPs L5 and L11 as tumour suppressors, they can also augment cell proliferation and migration once cells become transformed during tumorigenesis. For example, both RPs L11 and L5 can be overexpressed in a variety of cancerous cells [97,138,139,140]. Illustrating this view, RPL11 is more highly expressed in non-small cell lung carcinoma (NSCLC) cell lines compared to non-tumorigenic HBE cells [138]. This over expression has been shown to enhance cell proliferation, migration and cell cycle progression in the cell lines by upregulating *CCND1* (cyclin D1) and *CDK4* (cyclin-dependent kinase 4) expression [138]. Moreover, tumour cells often exhibit hyperactivated ribosome biogenesis (including increased RP production), which is necessary to support the very high translational requirements of cell proliferation within tumours. Supporting the important role of RPs in tumour cells, loss of RPS27a expression in lung adenocarcinoma cells promotes apoptosis via the RPL11-MDM2-p53 pathway [139].

Similarly, RPL5 is known to be overexpressed in colon cancer tissue compared to adjacent tissues [140]. It has been observed that RPL5 promotes progression of adenocarcinomas of the colon through activation of extracellular signal-regulated kinase (ERK), which phosphorylates itself and mitogen-activated protein kinase kinase (MEK). Additionally, RPL5 promotes the increased expression of c-Myc, ultimately contributing to tumour proliferation and migration. In some instances, it is the overexpression of mutated RPL5 proteins that promotes tumorigenesis and tumour progression [140]. A study utilizing the cancer genome atlas (TCGA) database for the analysis of somatic mutations in RP genes identified several mutations in RP genes as significant contributors to cancer incidence [101,141]. These studies revealed 139 RPL5 and 74 RPL11 heterozygous somatic mutations associated with cancer incidence. These mutations may block the normal tumour suppressive roles for RPL5 and RPL11 in cells (as described above). For example, the gene for RPL5 is located at a critical peak of heterozygous deletions on chromosome 1p22 [101]. A study of nearly 8000 patients with 16 different cancer types showed that RPL5 is one of the main RPs mutated in numerous cancers [141]. The above cancer-associated RPL5 and RPL11 mutations can impair the formation of the pre-ribosomal RPL5-RPL11-5S rRNA complex by disrupting the interaction between these RPs and the 5S rRNA [141]. Since the efficiency of RPL5 and RPL11’s ability to inhibit MDM2 from degrading p53 is enhanced only when both RPs are associated with the 5S rRNA [142], mutations in the genes encoding these two RPs may impair RPL5 and RPL11’s ability to augment cellular p53 levels. This would be expected to stimulate tumorigenesis and/or tumour progression.

DBA is a dominant autosomal bone marrow failure syndrome associated with mutations in the *RPL5* gene [143,144]. Since these mutations can also suppress RPL5-mediated p53 upregulation, it is not surprising that DBA patients have a higher risk of cancer incidence than the general population.

### 3.2. RPS3

RPS3 also has extra-ribosomal functions that impact tumorigenesis due to its role in DNA repair. Like many other DNA repair proteins, RPS3 is phosphorylated by Akt when cells experience DNA damage, after which it is translocated from the cytoplasm into the nucleus to take part in DNA repair activity. RPS3 has a high affinity towards 8-oxoguanine (8-oxoG), a common DNA lesion caused by ROS-mediated damage to the AP (apurinic/apyrimidinic) site of DNA [145]. Upon binding to these lesions, RPS3 acts as a negative regulator of non-homologous end joining (NHEJ) by binding to Ku heterodimers of the DNA-dependent protein kinase (DNA-PK) complex. This impairs error-prone NHEJ-mediated ligation reactions, preventing inappropriate gene deletions or insertions, and eventually promoting p53-dependent cell death [146]. Since RPS3 shares high sequence homology with the DNA ligase regulatory proteins XRCC4 and XLF, it has been suggested that RPS3 competes with XRCC4 and XLF for binding to DNA ligase IV [146]. This would help inhibit the NHEJ pathway.

While RPS3 binds to elongation factors 2 and 3 to promote protein translation [147], a study in PC12 cells suggests that the protein may also be a positive regulator of apoptosis [148]. The binding of RPS3 to E2F transcription factor 1 (E2F1) may act as an upstream factor of caspase-3. It has been observed that E2F1 can bind to the promoter region of the genes for the pro-apoptotic proteins Bim and Dp5, enhancing their expression. The enhanced expression of these proteins alters outer mitochondrial membrane permeability, resulting in the release of cytochrome C and the activation of apoptosis [148]. Furthermore, a study in glioblastoma cells revealed that upon knockout of ring finger protein 138, RPS3 was able to interact with DNA damage-inducible transcript 3 (DDIT3) to induce apoptosis when cells were irradiated [149]. Under conditions of genotoxic stress, RPS3 is also capable of sensitizing cells to apoptosis through its interaction with the tumour necrosis factor receptor type 1-associated death domain (TRADD), resulting in the activation of c-Jun N-terminal kinase (JNK) [150].

Interestingly, RPS3 has been shown to have yet another extra-ribosomal role. It has been shown that nerve growth factor (NGF) stimulation of PC12 cells promotes the phosphorylation of RPS3 by Akt, after which it translocates to the nucleus where it functions as an endonuclease. The increased accumulation of RPS3 in the nucleus facilitates sufficient DNA repair to ensure neuronal survival [148]. The phosphorylation of RPS3 also suppresses its above-described pro-apoptotic function [151]. Interestingly, this study reveals the dual role of RPS3 as both a pro-apoptotic and anti-apoptotic protein, depending upon cellular context [148,151,152]. Phosphorylation of RPS3 by Akt is necessary for it to bind to heat shock protein 90 (Hsp90). Binding of Hsp90 to RPS3 has been shown to prevent its ubiquitination and degradation by the proteasome, ensuring that RPS3 levels remain high enough to facilitate NHEJ-mediated DNA repair [153]. Moreover, the referenced study demonstrates that as ROS levels increase in HT1080 and HEK293T cells, RPS3 is transferred from Hsp90 to Hsp70. The dissociation of Hsp90 from the N-terminus of RPS3 exposes its mitochondrial targeting sequence, which is recognized by TOM70 (translocase of outer membrane 70), a protein that is located on the cytosolic side of the mitochondrial outer membrane. The RPS3-Hsp70-TOM70 complex guides RPS3 into the mitochondrion to facilitate DNA repair via its endonuclease activity. Experiments in human melanoma cell lines have also indicated that RPS3 has a role in stabilizing the Ca^2+^ gatekeeper mitochondrial calcium uptake 1 (MICU1) [154]. In doing so, S3 prevents an influx of Ca^2+^ in the mitochondrion and subsequent apoptosis [154].

Additional studies suggest that RPS3 can act as a tumour suppressor by other mechanisms. RPS3 is known to interact with nm23-H1 [155], a kinase that catalyzes the phosphorylation of nucleoside 5′-diphosphates to nucleoside 5′-triphosphates. This suppresses the metastasis of certain tumours [156]. The expression and activity of the nm23-H1 kinase is also known to reduce ERK signaling [157], which is necessary for the EMT associated with greater tumour cell invasion. Normally, tumour invasion occurs through the ability of matrix metalloproteinases (MMPs) to degrade the extracellular matrix and basement membrane. Nevertheless, in vitro and in vivo studies have revealed that RPS3 and nm23-H1 co-localize in cells at the cell periphery and in the cytoplasm. The association between these two proteins reduces MMP-9 secretion and ERK activation, thereby reducing cell invasive capability [155]. In contrast, mutations within the coding region for the RPS3 binding site of nm23-H1 inhibits nm23-H1’s interaction with RPS3, resulting in increased MMP-9 secretion and ERK activation in HT1080 cells [155].

In contrast to the above observations, other studies provide evidence that RPS3 can also play a role in tumour development and progression. Firstly, the overexpression of RPS3 in numerous cancers supports its involvement in tumorigenesis [158]. Whether this is the wildtype, phosphorylated, or mutated forms of RPS3 is unclear. Secondly, RPS3 is secreted and glycosylated, and it is the level of glycosylated RPS3 that regulates tumour cell invasion and migration [159]. Tumour cells also exhibit higher levels of glycosylated RPS3 than normal cells [159]. A small study of 73 patients with adenoid cystic carcinoma treated with cisplatin found that 37 patients had high tumour expression of RPS3 and this correlated with a higher incidence of tumour metastasis and poorer prognosis compared to patients with low tumour RPS3 expression [160].

Further supporting a role for RPS3 in tumour cell invasion and migration, siRNA-mediated reduction in RPS3 expression in BLB/C nude mice resulted in reduced adenoid cystic carcinoma migration, invasion, and cisplatin resistance [160]. Mass spectrometric analysis of tumours revealed that STAT1 (signal transducer and activator of transcription 1) is bound to RPS3 [160]. Interestingly, RPS3 acts as a non-canonical subunit of the NF-kB complex. Its interaction with P65 of the NF-kB complex induces transcriptional activation of genes associated with cell survival and epithelial stromal transformation [161]. NF-kB activity has been shown to be associated with increased expression of the ABCB1 drug efflux pump, facilitating drug resistance [162,163]. The above findings support the hypothesis that RPS3 is involved in the activation of STAT1/NF-kB signaling pathway through which it can promote cancer cell survival and chemoresistance. In breast cancers that are resistant to anthracyclines or taxanes, it was noted that the X-linked inhibitor of apoptosis (XIAP) is generally upregulated and overexpressed [164]. Typically, RPS3 binds to NF-kB and induces the expression of numerous anti-apoptotic genes [165]. However, in breast cancer cells it was found that knockdown of RPS3 downregulated cellular levels of XIAP at the protein level but not at the mRNA level. This suggests that RPS3 can regulate cellular levels of XIAP independently of NF-kB [166].

### 3.3. RPS6

RPS6 is an essential component of the 40S ribosomal subunit, playing a pivotal role in the translation of mRNA transcripts into protein. The *RPS6* gene is located on chromosome 9p21 and has a promoter region of high GpC content that lacks a consensus TATA box. It has been found that the *RPS6* gene is transcribed in mid-to-late G1 phase whereas rRNA genes are transcribed during the early G1 phase [167]. Studies in adult mouse livers revealed that knockout of *RPS6* disrupted the synthesis of the 40S ribosomal subunit in hepatocytes. This deficiency severely impaired the ability of hepatocytes to proliferate following partial hepatectomy, highlighting the critical role of RPS6 in liver regeneration. Similarly, the deletion of both alleles of RPS6 had profound consequences on organogenesis and immune system development, including severe defects in glandular development and a failure in T-cell maturation within the thymus [168]. These findings underscore the indispensable role of RPS6 in cellular processes that rely on active protein synthesis, such as tissue regeneration and immune cell development.

RPS6 was the first RP identified to undergo phosphorylation [169]. While S6 kinase1 (S6K1) is known to phosphorylate RPS6, other studies have revealed that numerous other protein kinases also phosphorylate RPS6 on residues Ser235 and Ser236. A recent study revealed that the C-terminus of RPS6 is highly phosphorylated at the beginning of mRNA translation and gradually becomes dephosphorylated as the elongation process continues, suggesting that the phosphorylation of RPS6 affects the translation of mRNAs based on their ORF length [170]. Supporting this view, RPS6 kinase S6K1 is known to interact with elFs [167]. These elongation factors remain bound to the ribosomes while they scan the 5′UTR of mRNAs towards their start codons. Subsequently, as the elongation progresses, the elFs are progressively released. The release of elFs (along with the release of S6K1) allows the RPS6 to become dephosphorylated as the peptide elongates [167]. Foot printing analysis also revealed that RPS6 phosphorylation promotes translation of mRNAs with shorter coding sequences than longer coding sequences since the phosphorylation of RPS6 progressively decreases with distance from the start codon [170].

Interestingly, the initiation and progression of NSCLC is strongly associated with the hyperphosphorylation of RPS6 by the Akt2 pathway [171]. In vitro experiments in human bronchial epithelial cells revealed that hyperphosphorylation of RPS6 was associated with increased cell proliferation and migration through enhanced cyclin (D1 and E), CDK (2 and 4), and migratory-related protein expression [171]. Hyperphosphorylation of RPS6 is significantly corelated with poor patient prognosis, particularly for stage 1 patients compared to patients with advanced disease [171]. RPS6 itself is also overexpressed in numerous human cancers and is associated with poor prognosis [102,171]. A recent study conducted in cholangiocarcinoma cells showed that overexpression of RPS6 is associated with tumour cell proliferation and silencing of RPS6 results in cell cycle arrest and reduced tumorigenicity [102]. The study also revealed that the knockdown of RPS6 significantly impacted cell cycle progression by downregulating the transcription of proteins essential for DNA replication. It is unclear whether the overexpressed protein was phosphorylated (or not).

Supporting the view that RPs have extra-ribosomal roles, RPS6 can be found in both the cytoplasm and nucleus of cells [87]. RPS6 has three putative nuclear localization signals (NLSs) and removal of the three signals results in the failure of its nuclear import [167]. RPS6 has been identified as a candidate RNA-binding protein (RNP) with structural similarity to the U1A protein [102]. Furthermore, mass spectrometric analysis revealed that RPS6 is associated with spliceosome complex proteins, indicating its potential role in RNA processing [102]. RPS6 appears to bind to multiple mRNAs, and the knockdown of RPS6 reduces expression of MCM complex proteins which are essential for the initiation and elongation phases of DNA replication during the cell cycle [102]. Supporting this view, a decrease in DNA replication was observed in cholangiocarcinoma cells upon silencing of RPS6 [102]. Moreover, RNA sequencing analysis demonstrated altered expression of 1088 genes upon RPS6 knockdown, including the downregulation of genes associated with DNA replication and cell cycle progression (CDK1, CDK2, MCM2, and UBC) [102].

A study of 12 glioma patients focused on the perivascular, perinecrotic and border regions of the tumours that predominantly harbor glioblastoma stem cells. Such regions revealed high expression of SOX2 and RPS6, both of which play critical roles in promoting tumour progression [172]. SOX2 maintains glioblastoma stem cell stemness, self-renewal, and therapy resistance while phosphorylated RPS6 is associated with the activation of the STAT3 signaling pathway [172]. Furthermore, sphere-forming assays demonstrated that siRNA-mediated reduction in RPS6 expression significantly suppressed the sphere forming potential of glioblastoma cells and strongly reduced the expression of glioblastoma stem cell markers. These findings highlight RPS6’s pivotal role in maintaining stem-like phenotypes and promoting glioblastoma cell proliferation, therapeutic resistance, and tumor recurrence.

### 3.4. RPL22

The LSU RPL22 consists of 128 amino acids. While it is primarily localized in the cytoplasm, it has also been observed to reside in the nucleus of cells. Despite RPL22 being incorporated into ribosomes, research studies revealed that knockout of RPL22 expression did not block protein synthesis, in part because the RPL22 knockout mice exhibited high incorporation of a RPL22-like protein (RPL22l1) into ribosomes [173]. Interestingly, a study in *Drosophila melanogaster* has shown that RPL22 can interact with chromatin components [174]. Immunofluorescence staining and chromatin immunoprecipitation analysis showed that both high histone H1 and RPL22 expression are associated with condensed, methylated, transcriptionally inactive chromatin. Overexpression of either H1 or RPL22 resulted in global suppression of a particular set of genes, while knockdown of either H1 or RPL22 results in an increase in the expression of the same gene set [174]. These observations provide strong support for the role of RPL22 in the epigenetic modification of genes. Further studies provided evidence of an interaction between RPL22 and the transposable element Doc5, which is known to be associated with condensed heterochromatin [175]. The study further showed that *Drosophila* RPL22 has an additional Ala-Lys-Pro rich sequence at its amino terminus that is the carboxy terminal region of histone H1. These findings suggest that *Drosophila* RPL22 has two functions: as an epigenetic modifier, and as a facilitator of protein translation.

As described in prior sections of this review, several RPs play a critical role in regulating the MDM2-p53 pathway. Like these RPs, L22 also has the ability to bind to MDM2 to prevent p53 ubiquitination [176]. RPL22 can also bind to mouse double minute 4 (MDM4) to regulate p53 activity in human H1299 NSCLC cells [177,178]. While MDM4, like MDM2, can bind to and inhibit p53, the former protein does not have intrinsic E3 ubiquitin ligase activity and dimerizes with MDM2 (reviewed in [179]). Moreover, the NSCLC cell studies also support a role for RPL22 in the regulation of transcript splicing [177,178]. One of the mechanisms that regulates MDM4 activity during stress is the skipping of exon 6 in the MDM4 pre-mRNA. This leads to the production of a shorter nonfunctional protein (MDM4-S) that fails to suppress p53 activity [177,178]. The knockdown of RPL22 decreases the expression of MDM4-S and increases the expression of full length MDM4 mRNA [178]. This suggests that RPL22 increases cellular p53 activity by altering the processing of MDM4 primary transcripts to favour higher levels of non-functional MDM4-S that cannot inhibit p53. This phenomenon was also seen in human HEK293T embryonic kidney cells (containing the SV40 T-antigen) [177,178].

RPL22 is also known to be associated with viral RNAs and proteins. A study in human B lymphocytes showed that upon infection with Epstein–Barr virus (EBV), RPL22 can translocate from the nucleus to the nucleoplasm and associate with the EBV-encoded RNA (EBER-1) [180]. Further studies in EBV infected cells showed that EBER-1 binds to the growth inhibitory and pro-apoptotic protein kinase R (PKR) and blocks its activation [181]. Subsequent studies have shown that RPL22 and EBER-1 have an antagonistic effect on each other’s ability to associate with PKR [180]. RPL22 can thus indirectly upregulate PKR activity by sequestering EBER-1.

Another study in NSCLC cells showed that RPL22 forms a complex with casein kinase 2α (CK2a) and directly inhibits CK2a substrate phosphorylation in a dose-dependent manner. Increased expression of CK2a was found to be associated with cell proliferation and growth in both normal and cancer cells, whereas down regulation of CK2a resulted in apoptosis [182]. All of the above studies strongly support a role for RPL22 as a tumour suppressor through multiple distinct mechanisms.

### 3.5. RPL27 and RPL27a

In tumour cells the LSU RPL27 can facilitate tumour growth and invasion. High RPL27 expression was found to promote colorectal tumour cell proliferation and stemness in vitro and in a mouse xenograft model via polo-like kinase 1 (PLK1) signalling. Moreover, silencing RPL27 in HCT116 and HT29 colorectal cancer cell lines decreased PLK1 expression, resulting in diminished cell division cycle 25C (CDC25C) phosphorylation and suppressed activation of the cell cycle regulators cyclin B1 and CDK1 [183]. These events correlated with reduced sphere-forming capacity, suggesting that RPL27 supports anchorage-independent growth in cells [183]. Further supporting the role of RPL27 in tumour cell proliferation, knockdown of RPL27 expression in SNU449 and HepG2 liver cancer cell lines resulted in increased expression of the pro-apoptotic proteins Bax and caspase-3 and decreased expression of the apoptosis inhibitor Bcl-2 [184].

RPL27a may also promote tumour cell invasion, since RPL27A was significantly higher in cells from a patient with metastatic breast cancer (MDA-MB-231 cells) than other breast tumour cell lines. Moreover, EIF2 signaling pathways were found to be activated in these cells. Knockdown of RPL27a expression in MDA-MB-231 breast tumour cells resulted in reduced cell migration and invasion [185]. Together, these observations suggest that RPL27a promotes cell migration, invasion, and possibly tumour metastasis through activation of the EIF2 signaling pathway.

### 3.6. RPL7a

RPL7a is a LSU RP that is encoded by SURF3, which is part of the surfeit gene cluster. It is also referred to as surfeit locus protein 3 (Surf3). RPL7a has 2 distinct RNA binding domains: one that binds its own mRNA with high efficiency and one that binds the 28S rRNA (with slightly lower efficiency) [186]. RPL7a also interacts with thyroid hormone receptors to inhibit their activities [187]. Interestingly, a portion of the RPL7A coding sequence can fuse with a segment of the trk proto-oncogene to form the chimeric oncogene trk-2h. Specifically, the 5′ region of trk-2h is derived from RPL7A and codes for a 41 amino acid sequence that fuses upstream of the coding sequence for the tyrosine kinase domain of a receptor for nerve growth factor (NTRK1) [188]. The chimeric gene produces a constitutively active 44 kDa tyrosine kinase that promotes anchorage-dependent growth in NIH3T3 fibroblasts—indicative of cell transformation [188]. NTRK1-related gene fusions are associated with many human tumours, including those of the breast, colon, lung and thyroid glands [189]. Interestingly, one study revealed that RPL7a can be significantly upregulated in colorectal cancer in the absence of the chimeric trk-2h oncogene [190].

Analyzing the influence of ethanol in human breast cancer cells, another study showed that ethanol modulates the expression of RPL7A transcripts in a dose- and time-dependent manner [191], presumably through estrogen-responsive elements within the RPL7A promoter. This likely results in increased expression of trk-2h transcripts, since the RPL7A promoter is fused upstream of NTRK1 sequences in the trk-2h gene fusion [188]. Moreover, ethanol promotes an elevation in plasma levels of estrogen, which can promote the proliferation of estrogen receptor-positive tumours [192]. Ethanol can thus act as a tumour promoter of trk-2h transformed tumour cells, increasing the proliferation of estrogen receptor-positive breast tumours. Ethanol can also induce a more aggressive cancer-associated fibroblast phenotype in these cells [193]. Further supporting a role for RPL7a in cancer, another study [194] using cannabidiol (CBD) identified key hub genes associated with triple negative breast cancer and RPL7A was among them.

### 3.7. RPL23 and RPL23a

RPL23 and RPL23a are distinct RPs having different sequences and whose transcripts stem from distinct chromosomal locations [195]. Similarly to other RPs, RPL23 can act as a tumour suppressor by inhibiting the E3 ubiquitin ligase MDM2, thereby promoting the accumulation of p53 in cells and consequently, p53-dependent cell-cycle arrest and apoptosis [196]. The binding of RPL23 with MDM2 has also been shown to occur simultaneously with RPs L5 and L11, effectively forming a quadruple complex [197]. In contrast, RPL23a appears to promote p53 degradation. RPL23 can promote tumour cell replication by negatively regulating Myc-interacting zinc finger protein 1 (Miz-1) dependent transcription of the cell cycle inhibitors P15^lnk4b^ and P21^cip1^ [198,199]. A study in myelodysplastic (MDS) patients showed that expression of RPL23 is much higher in high-risk MDS patients than low-risk MDS patients and this higher expression was associated with resistance to apoptosis and increased c-Myc expression [199]. In hepatocellular carcinoma cells, RPL23 was shown to be associated with the matrix metalloproteinase MMP-9, where it helps to stimulate cell invasion. Consistent with this view, the level of RPL23 transcripts in tumour biopsies from patients with hepatocellular carcinoma was considerably higher than that of adjacent liver tissues. Moreover, the level of RPL23 protein in hepatocellular carcinoma tissues positively correlated with tumour invasion, tumour vascularization, lung metastasis and poor survival [200].

### 3.8. RPL37

Like several RPs, RPL37 is also involved in the negative regulation of p53 through its association with MDM2 [112]. A Bioinformatics study on different RPs revealed that RPL37 is involved in the Wnt degradation pathway [201]. Through docking analysis, the study showed an association of RPL37 with the C-terminal tail of β-catenin, E-cadherin and APC, through which RPL37 inhibits tumour cell migration [201]. A tumour suppressive role for RPL37 is also supported by a retrospective study of 333 patients with non-metastatic locally advanced breast carcinoma. The study showed an increased risk of disease recurrence and death after neoadjuvant chemotherapy in patients with tumours having low RPL37 expression [202].

### 3.9. RPL3

Mutations in p53 are a major driver of tumour proliferation in many human cancers (reviewed in [203]). Further supporting a role for RPL3 in cancer, 5-FU or actinomycin D treatment of p53-mutated tumour cells was found to induce nucleolar stress, resulting in the release of RPL3 from ribosomes. The released RPL3 was then able to bind to SP1, suppressing SP1’s ability to promote the expression of cystathionine-β-synthase (CBS) [204,205]. Moreover, RPL3 can promote the translocation of CBS into mitochondria in these cells, where it promotes mitochondrial outer membrane permeabilization. This leads to the release of cytochrome C into the cytoplasm, activating caspase-9 and other downstream caspases to promote apoptosis [206]. In addition, RPL3 binding to Sp1 in p53-null lung and colon cancer cells promotes its ability to enhance transcription of the gene for the NF-kB inhibitor IκB. The elevated IB levels then block NF-κB’s translocation to the nucleus, preventing it from stimulating transcription of genes associated with cell survival [204]. Further supporting a role for RPL3 in cancer, RPL3 was found to induce apoptosis in p53-mutated Calu-6 lung cancer cells by promoting the overexpression of the pro-apoptotic protein Bax and the inhibition of the anti-apoptotic protein Bcl-2 [207]. These events also inhibit tumour cell migration and invasion [207]. Thus, RPL3 may be a useful target to overcome 5-FU resistance in patients with p53-mutated or p53-null tumours.

### 3.10. RACK1

RACK1 is considered to be an integral part of the 40S ribosomal subunit, where it serves as a platform for recruiting signal transduction molecules to the ribosome, particularly when cells are under stress [208]. RACK1 is also involved in IRES-mediated translation, which enables the expression of specific mRNA transcripts during stress or apoptosis [209]. A study in patients with oral squamous cell carcinoma revealed that RACK1 promotes oral squamous cell carcinoma progression, and its expression is negatively correlated with prognosis in these patients. Specifically, the study showed that RACK1 protein expression negatively correlated with the expression of interleukins 6 and 1 and chemokines 2 and 5. It also correlated with M2-like macrophage polarization via activation of the NF-κB pathway [210]. While the M2 macrophages are known for their anti-inflammatory and tissue repair functions, in the tumour microenvironment they enhance tumour growth and metastasis by suppressing anti-tumour immune responses and promoting angiogenesis. They do this by secreting cytokines IL-10 and TGF-β, which inhibit activation and proliferation of cytotoxic T cells and natural killer cells [211]. They also secrete VEGF, FGF-2, and MMP-9 to stimulate endothelial cell proliferation and new blood vessel formation [212]. In breast cancer cells, RACK1 binds to PSMD2 within the proteasome and this binding prevents proteasome-mediated degradation of ubiquitinated β-catenin, resulting in the activation of the Wnt signaling pathway and promotion of breast cancer proliferation [213].

Numerous studies reported that RACK1 is associated with EMT in cancer cells. RACK1’s interaction with key surface receptor proteins leads to the activation of a kinase associated with M2 macrophage polarity—a key event in stimulating cancer cell migration and invasion [214]. Further supporting the role of RACK1 with the EMT, depletion of RACK1 in glioblastoma cells reduced the expression of EMT markers [215].

## 4. Concluding Remarks

Dysregulation of cellular signalling pathways is a hallmark of cancer and leads to uncontrolled tumour cell survival, proliferation, metastasis, and drug resistance [2,216]. In normal cells, the energetically costly process of protein translation by ribosomes is tightly regulated, where ribosomes and their activity are tailored to meet cellular metabolic needs [217]. Moreover, as shown in this review, in addition to participating in the process of translating mRNA transcripts into proteins, RPs typically act as tumour suppressors by a variety of mechanisms. In contrast, tumour cells have dramatically altered signalling pathways that promote cell survival, cell proliferation, cell invasion, and altered cellular metabolism [218,219]. They also have tailored ribosomes that strongly support the high metabolic demands of tumours. Such “onco-ribosomes” result from epigenetic modifications, altered transcription, and processing defects in rRNAs [26,28]. In addition, cancer cells typically exhibit altered expression, mutations, or post-translational modifications of RPs that repress their typical tumour suppressive functions. Higher levels of unmutated or unmodified RPs may simply promote tumour progression in tumour cells by supporting their large translational and metabolic demands. The patterns of RP transcript expression also differ remarkably amongst tissues and tumours. In many cases, these differences correlate with molecular, pathological and clinical factors that impact on patient survival [220]. It remains unclear in many studies whether the RPs documented to be overexpressed in tumours are wildtype or mutated/post-translationally modified forms of the proteins (to block their efficacy as tumour suppressors). Nevertheless, the studies described in this review underscore the importance of the ribosome in cancer biology and the attractiveness of the ribosome as an anti-cancer target.

A number of current clinical trials are now assessing the utility of drugs targeting RNA polymerase I, rRNA splicing, rRNA modifications, and RPs to impact on a variety of human cancers, particularly in DNA mismatch repair-deficient tumours [221]. One of these agents (CX-5461) selectively inhibits RNA polymerase I (Pol I)-mediated transcription by disrupting the initiation stage of ribosomal RNA (rRNA) synthesis [222]. The drug has shown clinical benefit in patients with advanced hematologic cancers, including diffuse large B cell lymphoma, multiple myeloma, and anaplastic large cell lymphoma. The drug is also being assessed in clinical trials for patients with metastatic solid tumours harbouring homologous recombination deficiency mutations [222]. Due to off-target effects, CX-5461 can cause photosensitivity and hand–foot syndrome in patients [223]. This is likely due to effects of the drug on tissues with high protein translation needs (poor tissue specificity). Nevertheless, the drug received a fast-track designation by the Food and Drug Administration in the United States, due to its potential to target cancers with DNA repair defects [222]. A review on the development, utility and toxicities of recent drugs targeting ribosome biogenesis has recently been published [224].

**Figure 1:**
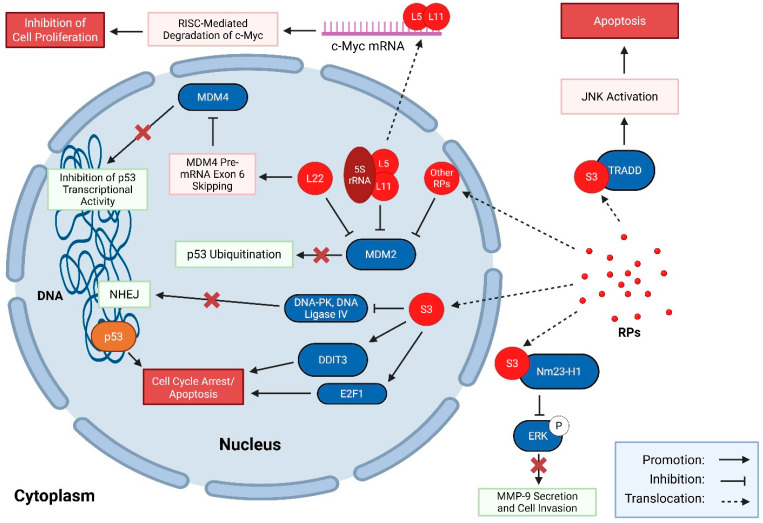
Illustrative examples of extra-ribosomal tumour-suppressive roles of RPs in mammalian cells under stress. Under normal conditions, excess RPs are rapidly degraded by a ubiquitin–proteasome system [84]. However, cellular stress can result in an accumulation of free RPs in the cell [88], which can then perform a variety of extra-ribosomal functions [85]. Several RPs, such as L5, L11, and L22 bind to the E3 ubiquitin ligase MDM2, thereby preventing p53 ubiquitination and subsequent degradation in the nucleus [108,124,176,197]. Through this interaction, cell cycle arrest mediated by p53 ensues [108,124,176,197]. Together, RPs L11 and L5 can also inhibit c-Myc-induced cell proliferation by recruiting RISC to c-Myc mRNA, resulting in its degradation [129,130]. L22 is capable of binding to MDM4 pre-mRNA, resulting in an alternative method of splicing, which skips exon 6 and creates the non-functional MDM4-S [177]. MDM4-S lacks the ability to block p53 from binding to its transcriptional targets, allowing p53 to suppress cell proliferation [177]. Lastly, S3 engages in multiple growth-suppressive pathways under various conditions of stress: it (a) interacts with DNA-PK and DNA ligase IV to negatively regulate NHEJ and promote apoptosis [146], (b) upregulates pro-apoptotic proteins by interacting with E2F1 [148] and DDIT3 [149], (c) enhances JNK pathway activity by associating with TRADD to increase cellular sensitivity to apoptosis [150], and (d) suppresses cell invasion by binding to nm23-H1 to reduce ERK phosphorylation and MMP-9 secretion [155].

**Figure 2:**
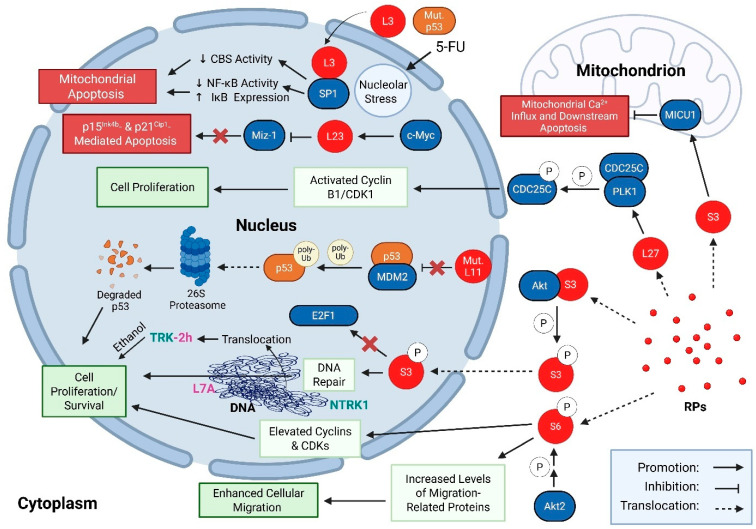
Illustrative examples of extra-ribosomal tumour-promoting roles of RPs within mammalian cells. Malignant cells are characterized by specific patterns of RP expression compared to normal cells [83]. Furthermore, mutations in RP genes have been identified in a variety of cancers [99]. Mutated forms of L11 (Mut. L11) within cancer cells have an impaired ability to bind MDM2, blocking L11’s inhibition of MDM2 E3 ubiquitin ligase activity [125]. This promotes the degradation of the tumour suppressor p53 by the 26S proteasome [111], promoting cell survival and proliferation. Phosphorylation of S3 by Akt promotes its endonuclease activity in the nucleus and also perturbs its pro-apoptotic interactions with E2F1, thereby promoting cell survival [148]. Furthermore, the expression of S3 in melanoma cancer cells has been found to stabilize levels of MICU1 within mitochondria in order to gatekeep Ca^2+^ entry and prevent apoptosis [154]. Hyperphosphorylation of S6 caused by downstream pathways of Akt2 has been associated with elevated levels of cyclins (D1 and E) and CDKs (2 and 4), promoting cell proliferation [171]. Additionally, S6 hyperphosphorylation has also been shown to enhance the migration of cells through increases in migratory-related proteins such as N-cadherin, vimentin, and MMP-2 [171]. Transcriptional activation of L23 by c-Myc leads to an inhibition of Miz-1, which normally activates the transcription of the cell cycle inhibitors p15 Ink4b and p21 Cip1 [199]. The suppression of p15^Ink4b^ and p21^Cip1^ may then confer resistance to apoptosis [199]. Expression of L27 has been found to activate PLK1-mediated phosphorylation of CDC25C, resulting in cyclin B1/CDK1 activation and nuclear entry followed by cellular proliferation [183,225]. Chromosomal instability in cancer cells can result in a translocation event where the ribosomal protein L7a gene (RPL7A) fuses with the gene for neurotrophic tyrosine kinase receptor 1 (NTRK1) to create a 44 kDa constitutively active protein that promotes uncontrolled cell proliferation, cell survival, and tumour formation [188]. Estrogen can act as a tumour promoter, increasing the proliferation and invasion of trk-2h transformed cells [191,193]. In contrast, treatment of p53-mutated cancer cells with certain chemotherapy agents can block tumour promotion through the activity of RPs. For example, 5-FU promotes nucleolar stress in p53-deficient tumour cells, resulting in the release of RPL3 from the ribosome, where it interacts with the transcription factor Sp1 in the nucleus, increasing its activity. The elevated Sp1 activity increases the expression of IκB, which inhibits NF-kB-dependent survival pathways and promotes mitochondrial apoptosis [207]. The formation of the L3/Sp1 complex also reduces expression and activity of cystathionine-κ synthase (CSB), an enzyme that would otherwise promote H2S production, cancer cell bioenergetics, and survival [205]. This results in mitochondrial apoptosis and underscores the utility of 5-FU in the treatment of some p53-mutated cancers.

Tabular and illustrative summaries of the extra-ribosomal tumour suppressive roles for RPs in wildtype tissues can be found in Table 1 and Figure 1, respectively, while similar tabular and illustrative summaries of the extra-ribosomal tumour-promoting roles for RPs are summarized in Table 2 and Figure 2, respectively.

**Table 1:** Extra-ribosomal Tumour Suppressive Roles for Select RPs.

Ribosomal Protein	Tissue	Role	Effect on Cancer	References
RPL5	Breast	Tumour suppressor	Modulates ER stress and autophagy via regulating E2F1	[226]
RPL5	Glioblastoma (GBM)	Tumour suppressor	Associated with PTEN protein suppression	[141]
RPL5	NSCLC	Tumour suppression	Down-regulates EMT markers	[227]
RPL5 & L11	Lung, Breast	Tumour suppressor	Destabilizes c-Myc	[137,138]
RPL3	Lung, Colon	Tumour suppressor	Induces apoptosis	[205,207]
RP- L5, L11, L23, S3, S7, S14, L26, S19, S20, L22, S27L, L37, S15, S27, S26	Liver, Gastric, GBM, Skin, Lung, Breast	Stabilizing p53 by preventing its degradation by MDM2	Cell cycle arrest	[106,107,108,110,117,196]
RPL22	Retina, Kidney, NSCLC	Tumour suppression	Regulates p53 levels and induces apoptosis	[182,228]
RPS3	GBM	Pro-apoptotic	Interact with DDIT3 (CHOP)	[149]

**Table 2:** Extra-ribosomal Tumour Initiation and Promotion Roles for Select RPs.

Ribosomal Protein	Tissue	Role	Effect on Cancer	Reference
RPS3	Fibroblast, Kidney, Breast, Melanoma	Oncogenic	DNA repair, inhibits apoptosis	[152,154,229,230]
RPS3	NSCLC	Radioresistance	Interacts with NF-kB	[231]
RPS7	Prostrate	Tumour proliferation	Associated with EMT markers	[232]
RPS13, L23	Gastric	Tumour proliferation	Drug resistance	[233,234]
RPS6	GBM	Tumour stemness and t sphere formation	Regulates STAT3 expression	[172]
RPS6	Liver	Tumour Proliferation	Regulates AKT/mTOR signalling	[171]
RPS6	Oral squamous, cervical, renal, NSCLC, liver	Tumour proliferation	Phosphorylated RPS6 activates expression of cyclins	[167,171,235,236,237,238]
RPL7a	Breast, Colon, Lung, Thyroid	Tumour Proliferation, Invasion	Formation of trk-2h oncogene	[188]
RPL23	Blood, liver	Tumour proliferation	Apoptotic resistance, increases MMP protein expression	[199,239]
RPL27	Colorectal	Tumour proliferation	Interacts with PLK1	[183]

## Abbreviations

5′ UTR	5′ untranslated region
CBS	cystathionine-b-synthase
CCND1	cyclin D1
CDK	cyclin-dependent kinase
CK2a	casein kinase 2 alpha
cMYC	cellular Myc (gene associated with myelocytomatosis in birds)
DBA	Diamond-Blackfan anemia
DDIT3	DNA damage-inducible transcript 3
DNA-PK	DNA-dependent protein kinase
E2F1	E2F transcription factor 1
EBER-1	EBV-encoded RNA 1
EBV	Epstein–Barr Virus
EIF2	eucharyotic initiation factor 2
EMT	epithelial–mesenchymal transition
ERK	extracellular signal-related kinase
FBL	fibrillarin
GBM	glioblastoma
HSP	heat shock protein
IRES	internal ribosome entry site
JNK	c-Jun N-terminal kinase
LSU	large subunit of the ribosome
MDM	mouse double minute
MeCP2	methyl-CpG-binding protein
MEK	mitogen-activated protein kinase kinase
miRNA	micro ribonucleic acid
MMP	matrix metalloproteinase
mRNA	messenger RNA
mTOR	molecular target of rapamycin
NGD	no-go decay
NHEJ	non-homologous end joining
NMD	nonsense-mediated decay
NRD	non-functional RNA decay
NSCLC	non-small cell lung cancer
NSD	non-stop decay
p53	53 kDa tumour suppressor protein
RACK1	receptor for activated kinase 1
RISC	RNA-induced silencing complex
RNA	ribonucleic acid
ROS	reactive oxygen species
RP	ribosomal protein
RQC	ribosome quality control
rRNA	ribosomal ribonucleic acid
SSU	small subunit of the ribosome
STAT	signal transducer and activator of transcription
Surf3	surfeit locus protein 3
Tap73	transactivation isoform of p73
tRNA	transfer ribonucleic acid
Ub	ubiquitin
UPS	Ubiquitin–proteasome system
XIAP	X-linked inhibitor of apoptosis
